# Facteurs de risque du décollement de rétine au Togo

**DOI:** 10.11604/pamj.2017.28.74.13418

**Published:** 2017-09-26

**Authors:** Nidain Maneh, Danièle Christelle Tchapbou Moyou, Kassoula Batomaguela Nonon Saa, Messan Kokou Amedome, Kossi Dzidzinyo, Bénédicte Marèbe Diatewa, Koffi Didier Ayena, Mèba Banla, Komi Patrice Balo

**Affiliations:** 1 Université de Lomé, FSS, Service d’Ophtalmologie Chu-Campus de Lomé, Togo; 2 Service d’Ophtalmologie Chu-Campus de Lomé, Togo; 3 Université de Lomé, FSS, Service d’Ophtalmologie Chr Sokodé, Togo; 4 Université de Lomé, FSS, Service d’Ophtalmologie CHU-Kara Togo; 5 Université de Lomé, FSS, Service d’Ophtalmologie CHU Sylvanus Olympio Lomé-Togo; 6 Université de Lomé, FSS, Service d’Ophtalmologie Hôpital de Bè

**Keywords:** Décollement, rétine, facteurs de risque, Togo, Detachment, retina, risk factors, Togo

## Abstract

**Introduction:**

le décollement de rétine pose un problème de prise en charge dans les pays en développement par manque de plateau technique. Sa prévention passe par la connaissance et l'éviction de ses facteurs de risque. Le but de l'étude était d'identifier les facteurs de risque du décollement de rétine chez le Togolais. Méthode: il s'est agi d'une étude rétrospective et descriptive, réalisée dans le service d'ophtalmologie du Chu-Campus et dans un cabinet privé d'ophtalmologie de Lomé du 2 Janvier 2011 à 31 Décembre 2015. Ont été inclus dans l'étude les dossiers des patients portant le diagnostic de décollement de rétine. Le diagnostic de décollement de rétine avait été retenu devant: la présence d'un décollement de rétine à l'examen du fond d'œil ou à l'échographie oculaire.

**Méthodes:**

il s'est agi d'une étude rétrospective et descriptive, réalisée dans le service d'ophtalmologie du Chu-Campus et dans un cabinet privé d'ophtalmologie de Lomé du 2 Janvier 2011 à 31 Décembre 2015. Ont été inclus dans l'étude les dossiers des patients portant le diagnostic de décollement de rétine. Le diagnostic de décollement de rétine avait été retenu devant: la présence d'un décollement de rétine à l'examen du fond d'œil ou à l'échographie oculaire.

**Résultats:**

au total, 116 yeux de 100 patients avaient un décollement de rétine dont 40 à l'OD, 44 à l'OG et 16 bilatéraux (32yeux). L'âge moyen des patients était de 46,65 ± 16,46 ans [07 ans ; 87 ans], une prédominance masculine et avec un sex-ratio = 0,32 (F/H). Les diabétiques représentaient 17% et les patients drépanocytaires 16%. Les patients myopes représentaient 5%, les yeux pseudophaques représentaient 17,2% et les yeux aphaques 3,4%. Quatre déchirures rétiniennes (14,28 % des DR rhegmatogènes) étaient retrouvées dont 2 déchirures en supéro-temporal, une déchirure en inféro-nasal et une déchirure en inféro-temporal. Le décollement était total pour 35 yeux (52,2%) et partiel pour 24 yeux (35,8%). Vingt yeux présentaient des proliférations vitréo-rétiniennes, 5 yeux avaient un vitré hémorragique et 6 yeux une hyalite. Le diabète et la drépanocytose étaient les facteurs de risque du DR tractionnel (p=0,006 et p=0,0003), et la chirurgie de cataracte le facteur de risque du DR rhegmatogène (p=0,0097).

**Conclusion:**

le diabète, la drépanocytose et la chirurgie oculaire étaient les facteurs de risque les plus importants du DR. Une meilleure prise en charge de ces pathologies et une maîtrise de la chirurgie de la cataracte par l'ophtalmologiste préviendraient le décollement de rétine.

## Abstract (en)

**Introduction:**

Retinal detachment poses management problems in developing countries due to the shortage of technical equipment. Prevention passes through knowledge and elimination of risk factors. This study aimed to identify the risk factors for retinal detachment in people living in Togo.

**Methods:**

We conducted a retrospective and descriptive study in the Department of Ophthalmology at the Chu-Campus and in an ophthalmology office in Lomé from 2 January 2011 to 31 December 2015. The medical records of patients with retinal detachment were included in the study. The diagnosis of retinal detachment was confirmed based on funduscopic exam or ocular ultrasound.

**Results:**

in total, 116 eyes of 100 patients had retinal detachment of whom 40 in the RE, 44 in the LE and 16 bilaterally. The average age of patients was 46.65 ± 16.46 years [07 years; 87 years], with a male predominance and with a sex-ratio = 0.32 (W/M). Diabetic patients accounted for 17% and patients with sickle cell disease accounted for 16%. Myopic patients accounted for 5%, pseudophakic patients accounted for 17.2% and aphakic patients accounted for 3.4%. Four patients had retinal tear (14.28% of rhegmatogenous detachment) including 2 superotemporal tears, 1 inferior nasal tear and 1 inferior temporala tear. 35 eyes (52.2%) had total retinal detachment while 24 eyes (35.8%) had partial retinal detachment. Twenty eyes had vitreoretinal proliferations, 5 eyes had itreous hemorrhage and 6 eyes had hyalite. Diabetes and sickle cell disease were the risk factors for tractional RD (p=0.006 and p=0.0003) while cataract surgery was the risk factor for rhegmatogenous RD (p=0.0097).

**Conclusion:**

Diabetes, sickle cell anemia and eye surgery were the major risk factors for RD. A better management of these pathologies and ophthalmologist’s mastery of cataract surgery may prevent retinal detachment.

## Introduction

Le décollement de rétine (DR) est le clivage entre la neurorétine et l'épithélium pigmenté de la rétine. Il peut être causé par une brèche de l'épaisseur de la rétine (décollement rhegmatogène) [1], par une traction directe sur la rétine par le vitré ou un tissu fibreux rétractile (DR tractionnel), ou par une pathologie vasculaire ou choroïdienne plus rarement (DR exsudatif). La littérature rapporte une incidence moyenne du décollement de rétine dans la population mondiale de 6 à 18 pour cent mille habitants [2]. L'incidence du décollement de rétine n'est pas bien connue en Afrique. Plusieurs facteurs de risque généraux et oculaires sont incriminés dans la genèse du décollement de rétine. Son traitement qui fait appel à une unité de chirurgie vitréo-rétinienne est très peu accessible dans les pays en développement. C'est dans cette optique que nous réalisions cette étude préliminaire qui avait pour objectif de rapporter la fréquence du décollement de rétine et d'identifier les facteurs de risque chez le noir Togolais en vue de sa prévention.

## Méthodes

Il s'est agi d'une étude rétrospective et descriptive portant sur la fréquence et les facteurs de risque du décollement de rétine allant du 2 Janvier 2011 au 31 Décembre 2015. Cent dossiers des patients de tout âge ayant consulté au Chu-Campus et dans un cabinet privé d'ophtalmologie de Lomé et chez lesquels le diagnostic de décollement de rétine a été posé sur la base de l'examen du fond d'œil (ou de l'échographie oculaire étaient inclus. Tous les dossiers incomplets ne comportant pas l'âge, le sexe, la description de l'examen du fond œil n'étaient pas inclus. L'âge, le sexe, les antécédents l'état du segment antérieur et postérieur (état du vitré, proliférations vitré-orétiniennes, topographie de la déchirure, type du DR), étaient étudiés. Les données collectées ont été traitées avec le logiciel épi info Version 3.5.4. et la comparaison des variables a été faite à l'aide du test de Chi2; le seuil de significativité était retenu pour p<0,05.

## Résultats

Pendant le période d'étude un total de 110 dossiers des patients présentant un décollement de rétine (DR) sur les 30863 consultants. Parmi lesquels cent (0,32%) dossiers de patients (116 yeux) respectaient nos critères d'inclusion dont 40 patients avec un DR unilatéral droit, 44 avec un DR unilatéral gauche et 16 avec un DR bilatéral (32 yeux).

**Sexe et âge:** on notait une prédominance masculine avec 76 hommes et 24 femmes soit un sex-ratio = 3,16 (H/F). L'âge moyen des patients était de 46,65 ± 16,46 ans [07 ans et 87 ans]. La tranche d'âge de [40-60ans[ était la plus représentée avec 41,0% (**Tableau 1**).

**Tableau 1:** Répartition des patients selon l’âge

	Effectif	Pourcentage (%)
**] 0 - 20[**	6	6
**[20 - 40[**	29	29
**[40 - 60[**	41	41
**≥ 60 ans**	24	24
**TOTAL**	100	100,0

**Antécédents généraux et personnels:** pour les antécédents généraux 17% des patients étaient diabétiques, 18% étaient drépanocytaires dont 15 présentaient la forme SC, un la forme SS et deux patients portaient un trait drépanocytaire AS (**Tableau 2**). La chirurgie oculaire était la plus représentée avec 25% dont 21(21%) de phaco-exérèse et 2 (2%) de chirurgie du DR. Cinq cas de myopie ont été retrouvés dont 2 myopies fortes de 8 et 10 dioptries (**Tableau 2**).

**Tableau 2:** Répartition des patients selon les antécédents personnels

	Effectif	%
**Antécédents personnels généraux**		
Diabète	17	17,0
Drépanocytose	18	18,0
HTA	16	16,0
**Antécédents personnels ophtalmologiques**		
Chirurgie oculaire	25	25,0
Myopie	5	5,0
Traumatisme oculaire	12	12,0

**Examen du fond de l'œil:** nous avons retrouvé 67 yeux pour lesquels le diagnostic de DR a été fait au fond d'œil. Ainsi, il y avait 27 DR à l'œil droit (OD), 28 DR à l'œil gauche (OG) et 6 DR bilatéraux (12yeux). Cinq yeux sur les 11 yeux présentant un décollement de rétine avaient un vitré hémorragique et 6 yeux une hyalite. Vingt yeux présentaient des proliférations Vitréo-rétiniennes. Quatre déchirures rétiniennes (14,28 % des DR rhegmatogènes) étaient retrouvées dont 2 déchirures en supéro-temporal, une déchirure en inféro-nasal et une déchirure en inféro-temporal. Le décollement de rétine était total pour 35 yeux (52,2%) et partiel pour 24 yeux (35,8%). Pour les décollements partiels, la macula était soulevée dans 16 yeux (66,6%). Chez 71 yeux la nature du décollement avait été précisée dont 28 patients (39,4%) avaient un DR rhegmatogène et 28 (39,4%) patients avec un DR tractionnel (**Tableau 3**).

**Tableau 3:** Répartition des yeux selon la nature du décollement de rétine (DR)

	Effectif	%
DR rhegmatogène	28	39,4
DR tractionnel	28	39,4
DR exsudatif	8	11,3
DR mixte	7	9,9
**TOTAL**	**71**	**100,0**

**Antécédents et type de décollement:** la nature du DR avait été précisée chez 71 patients. Le DR tractionnel a été prédominant chez les patients diabétiques (p=0,006) et drépanocytaires (p=0,0003) (**Tableau 4**). Des 116 yeux avec un décollement de rétine, nous avons retrouvé 20 (17,2%) yeux pseudophaques et 4 (3,4%) aphaques. Pour les 24 patients ayant bénéficié d'une chirurgie oculaire, 15 patients avaient un DR rhegmatogène (p=0,0097) et 6 un décollement mixte (**Tableau 5**).

**Tableau 4:** Répartition des antécédents personnels généraux selon la nature du décollement de rétine (DR)

	Diabète			Drépanocytose			HTA		
	Oui	Non	p	Oui	Non	P	oui	non	p
**DR rhegmatogène**	1	27	0,0030	0	28	-	2	26	0,065
**DR tractionnel**	12	16	**0,006**	13	15	0,0003	9	19	0,069
**DR exsudatif**	1	7	0,7147	0	8	-	2	6	0,941
**DR mixte**	3	4	0,4421	3	4	0,3794	1	6	0,904

**Tableau 5:** Répartition des antécédents personnels ophtalmologiques en fonction de la nature du décollement de rétine (DR)

	Chirurgie oculaire			Myopie			Traumatisme oculaire		
	Oui	Non	p	Oui	Non	p	oui	Non	p
DR rhegmatogène	15	13	**0,009**	3	1	0,7	8	1	0,09
DR tractionnel	3	25	0,001	1	1	0,5	1	3	0,14
DR exsudatif	0	8	0,083	0	0	0,0	0	0	0,00
DR mixte	6	1	0,003	0	1	0,8	1	1	0,78

## Discussion

Le présent travail présente une limite propre aux études rétrospectives liées à l'insuffisance de description dans les dossiers de certaines données de l'examen. De rares études prospectives ont été réalisées en Afrique [3], notre étude qui est une étude rétrospective préliminaire malgré ces limites, a apporté les premières données sur le décollement de rétine au Togo. L'âge moyen était de 46,65 ± 16,46 ans [7 ans et 87 ans] et la tranche de 40 à 60 ans, était plus représentée 41%. Comme le rapportent des études dans les pays en développement, le décollement de rétine survient souvent chez un adulte jeune [3,4]. Une prédominance masculine était retrouvée, Seck et al. [5] au Sénégal ainsi que Nwosu et al. [6] au Nigéria ont également fait le même constat. Nous avons retrouvé un lien statistiquement significatif entre la présence du diabète, de la drépanocytose et le décollement de rétine tractionnel (p=0,006 et p=0,0003). Le diabète et la drépanocytose sont donc des facteurs de risque retrouvés dans le décollement de rétine surtout tractionnel [7]. Les déchirures ont constitué 14,28 % des DR rhegmatogènes ce qui est proche de celles retrouvées en Afrique du sud (15,7%) [8]. Des taux supérieurs aux nôtres ont été rapportés en Ethiopie (86,2%) [3], au Nigéria (54,8%) [6]. En effet des taux allant de 2,5% à 22% de déchirures non vues dans les décollements rhegmatogènes des pseudophaques sont rapportés [9–11]. Malgré le nombre réduit de déchirures retrouvées, le quadrant supéro-temporal est la localisation prépondérante comme dans certaines études [3,6]. Vingt-un pourcent des patients ayant bénéficié d'une chirurgie de la cataracte ont présenté un DR à l'œil opéré et 2% d'une chirurgie du décollement de rétine à l'œil controlatéral. Des 116 yeux présentant un décollement de rétine, nous avons retrouvé 17,2% (20) d'yeux pseudophaques et 3,4% (4) d'aphaques. Des résultats supérieurs aux nôtres ont été retrouvés par Farzan et al. [12] en Iran avec 32,2% des patients avec antécédents de chirurgie de cataracte dont 24,1% étaient pseudophaques et 8,1% aphaques. Chez Nwosu au Nigéria, la chirurgie intraoculaire et la chirurgie de la cataracte étaient le principal facteur de risque chez 35,5% des patients [6]. En Taiwan, 11,6% des patients avaient des antécédents de chirurgie de la cataracte [13]. Dans notre serie, nous avons retrouvé une correlation entre la présence d'une chirurgie oculaire et le décollement de rétine rhegmatogène (p= 0,0097). La chirurgie de la cataracte est connue comme facteur de risque du décollement de rétine rhegmatogène si celle-ci se complique de rupture de la capsule postérieure et d'issue de vitré. Pour une prévention du decollement de rétine après phaco-excérèse, il impose aux ophtalmologistes une maitrise et une amélioration des techniques de la chirurgie de la cataracte afin de réduire la rupture capsulaire postrérieure et l'aphakie. Nous avions retrouvé 5% de patients myopes dont 2% présentait une myopie forte. Ces résultats sont inférieurs à ceux retrouvés par Nwosu au Nigéria, avec 7,1% de myopie forte [6]. En Taiwan, San-Ni Chen a noté 10,51% de myopes forts [13]. Nos résultats étaient inférieurs autres études car sous-estimés du fait de l'absence de la réfraction des patients sur certains dossiers. Une correction optique précoce et un suivi régulier par l'examen du fond de l'œil des myopes permettraient de prendre en charge les signes précurseurs rétiniens de décollement (trous atrophiques). Les antécédents de traumatismes oculaires représentaient 10% des patients. Contrairement des taux plus élevés entre 14,8% et 34% ont été rapportés [3,12] faisant du traumatisme oculaire un facteur de risque non négligeable du décollement de rétine. Toutefois l'éducation des jeunes contre les jeux violents, la sensibilisation au port des lunettes de protection dans les métiers à risque permettront de réduire le taux de décollement de rétine partraumatismes oculaires.

## Conclusion

De cette étude il en ressort que les adultes jeunes et les sujets de sexe masculin étaient plus atteints du décollement de rétine. Le diabète et la drépanocytose constituaient les facteurs de risque prépondérant du décollement de rétine tractionnel d'où la nécessité d'une meilleure prise en charge de ces pathologies. La chirurgie de la cataracte, facteur de risque important du décollement de la rétine rhegmatogène, doit être maîtrisée par l'ophtalmologiste pour prévenir le décollement de rétine du pseudophaque et de l'aphaque.

### Etat des connaissances actuelles sur le sujet

- La myopie forte (>6 D) est un facteur de risque important de décollement de rétine;
- Chirurgie intraoculaire surtout extraction intracapsulaire de la cataracte est un facteur de risque du décollement de rétine.

### Contribution de notre étude à la connaissance

- Le sexe masculin est un facteur de risque du décollement de rétine;
- Diabète et drépanocytose étaient des facteurs de risque de décollement de rétine;
- Malgré l'introduction de la chirurgie de la cataracte à petite incision manuelle, elle est pourvoyeuse de décollement de la rétine rhegmatogène.

## Conflits d’intérêts

Les auteurs ne déclarent aucun conflit d'intérêts.
